# 5-Hydroxytryptamine (5-HT) Cellular Sequestration during Chronic Exposure Delays 5-HT_3_ Receptor Resensitization due to Its Subsequent Release*

**DOI:** 10.1074/jbc.M114.594796

**Published:** 2014-10-03

**Authors:** J. Daniel Hothersall, Amy Alexander, Andrew J. Samson, Christopher Moffat, Karen A. Bollan, Christopher N. Connolly

**Affiliations:** From the Medical Research Institute, University of Dundee, Dundee DD1 9SY, Scotland, United Kingdom

**Keywords:** Intestine, Ion Channel, Membrane Trafficking, Pharmacology, Receptor Desensitization, Receptor Internalization, Selective Serotonin Reuptake Inhibitor (SSRI), Serotonin, Serotonin Transporter, 5-HT_3_

## Abstract

The serotonergic synapse is dynamically regulated by serotonin (5-hydroxytryptamine (5-HT)) with elevated levels leading to the down-regulation of the serotonin transporter and a variety of 5-HT receptors, including the 5-HT type-3 (5-HT_3_) receptors. We report that recombinantly expressed 5-HT_3_ receptor binding sites are reduced by chronic exposure to 5-HT (IC_50_ of 154.0 ± 45.7 μm, *t*_½_ = 28.6 min). This is confirmed for 5-HT_3_ receptor-induced contractions in the guinea pig ileum, which are down-regulated after chronic, but not acute, exposure to 5-HT. The loss of receptor function does not involve endocytosis, and surface receptor levels are unaltered. The rate and extent of down-regulation is potentiated by serotonin transporter function (IC_50_ of 2.3 ± 1.0 μm, *t*_½_ = 3.4 min). Interestingly, the level of 5-HT uptake correlates with the extent of down-regulation. Using TX-114 extraction, we find that accumulated 5-HT remains soluble and not membrane-bound. This cytoplasmically sequestered 5-HT is readily releasable from both COS-7 cells and the guinea pig ileum. Moreover, the 5-HT level released is sufficient to prevent recovery from receptor desensitization in the guinea pig ileum. Together, these findings suggest the existence of a novel mechanism of down-regulation where the chronic release of sequestered 5-HT prolongs receptor desensitization.

## Teaser

**Background:** Altered levels of 5-HT are thought to contribute to cognitive and gastrointestinal dysfunction.

**Results:** Intracellular accumulation of 5-HT leads to a prolonged loss of 5-HT_3_ receptor function.

**Conclusion:** 5-HT_3_ receptor desensitization is maintained by a chronic release of 5-HT.

**Significance:** Prolonged 5-HT_3_ receptor dysfunction may result from the clinical use of selective serotonin reuptake inhibitors or pathologically raised 5-HT levels.

## Introduction

The 5-hydroxytryptamine type-3 (5-HT_3_)^2^ receptor is a cationic ligand-gated ion channel that mediates fast excitatory responses to serotonin (5-HT) in both the central and peripheral nervous systems. The 5-HT_3_ receptors belong to the Cys-loop superfamily of ligand-gated ion channels that includes the nicotinic acetylcholine, γ-aminobutyric acid type A and glycine receptors (1). In humans there are five 5-HT_3_ receptor subtypes (5-HT3A-E), with homomeric 5-HT_3_A and heteromeric 5-HT_3_AB being the most commonly expressed and best characterized (2).

5-HT_3_ receptors are found in many regions of the central nervous system including the hippocampus, entorhinal cortex, and frontal cortex (3). However, the highest level of 5-HT found *in vivo* is located in the enteric nervous system, where serotonergic signaling through 5-HT_3_ receptors regulates important physiological functions such as gut pacemaker activity, motor activity, luminal sensing, and intestinal secretion (4, 5). To date the therapeutic value of 5-HT_3_ receptor ligands is limited to the use of antagonists in the control of chemotherapy-, radiotherapy- and surgery-induced emesis (6, 7) and irritable bowel syndrome (8). Serotonergic neurons express the serotonin transporter (SERT) (9), which terminates receptor signaling by the efficient removal of extracellular 5-HT.

The serotonergic synapse is dynamically regulated, and elevated 5-HT has been reported to cause a down-regulation of the function of SERT (10, 11), 5-HT_1A_ (12), 5-HT_2A_ (13), 5-HT_2C_ (14), 5-HT_4_ (15), and 5-HT_7_ (16) receptors. In the case of 5-HT_3_ receptors, the long term down-regulation of receptor function/expression has been reported after chronic exposure to ligands (17–21). In the case of agonist-induced down-regulation, this has been proposed to occur by receptor internalization (17, 18), whereas antagonist-induced down-regulation is reported to occur by prolonged binding (22, 23) rather than internalization (24).

As discussed, elevated levels of 5-HT may alter serotonergic signaling by disrupting the function or inducing the internalization of many components of serotonergic neurotransmission. A number of pathophysiological disorders are characterized by elevated 5-HT levels, such as diarrhea-predominant irritable bowel syndrome, chemo/radiotherapy, coronary artery disease (25), and complex regional pain syndrome (26). Similarly, the therapeutic use of selective serotonin reuptake inhibitors or a Western-style high fat/calorie diet are also characterized by elevated 5-HT (27).

We, therefore, sought to characterize how 5-HT_3_ receptors are regulated by chronic exposure to 5-HT. In keeping with the previous studies (17, 19, 21), we find that the chronic exposure to high concentrations of 5-HT decreases the number of available 5-HT_3_ receptor binding sites without altering surface receptor levels and is not blocked by inhibitors of endocytosis, indicating that receptor internalization is not required for agonist-induced down-regulation as observed for antagonist-induced down-regulation (22). The 5-HT-induced down-regulation is potentiated (67-fold) by SERT, highlighting a role for the intracellular transport of 5-HT. Sequestered 5-HT is released slowly from both COS-7 cells and guinea pig ileum. In support of a role for the slow 5-HT release, the resensitization of 5-HT_3_ receptor-mediated contractions in the intact guinea pig ileum is inhibited by low (5 μm) 5-HT.

## EXPERIMENTAL PROCEDURES

### 

#### 

##### Chemical and Drug Sources

5-Hydroxytryptamine hydrochloride (5-HT, serotonin), nystatin, dynasore hydrate, brefeldin A, filipin, TX-114, digitonin anti-HA/myc antibodies (Sigma), 2-methyl-5-hydroxytryptamine hydrochloride (Tocris, Bristol, UK), [^3^H]BRL-43964 ([^3^H]granisetron), and 5-[^3^H]HT (PerkinElmer Life Sciences). Cell culture reagents, Amplex UltraRed, and Alexa Fluor 488/568 conjugated anti-mouse antibody (Invitrogen). Horseradish peroxidase conjugated anti-mouse antibody (GE Healthcare).

##### Cell Culture and Transfection

Simian COS-7 cells (ACC CRL 1651) were maintained in DMEM supplemented with 10% fetal bovine serum, 2 mm l-glutamine, 1 mm sodium pyruvate, 100 μg/ml streptomycin, and 100 units/ml penicillin in an atmosphere of 5% CO_2_ at 37 °C. Exponentially growing cells were transfected by electroporation (400 V, ∞Ω, 125 microfarads; Bio-Rad Gene Electropulser II). 10 μg of DNA was used per transfection (2 × 10^6^ cells). Cells were analyzed 24–48 h after transfection. Human 5-HT_3A_-myc was expressed from the mammalian expression vector PRK5JD (28). Human SERT in pcDNA3.1 was a kind gift from Beate Niesler (University of Heidelberg, Heidelberg, Germany) and subcloned into PRK5JD.

##### Radioligand Binding

[^3^H]Granisetron (specific binding = 83.1 Ci/mmol) binding was performed on intact COS-7 cells cultured on poly-l-lysine-coated 96-well plates. These methods selectively measure cell surface 5-HT_3_ receptor binding sites (22). Mock-transfected cells were used to determine the background. Cells were incubated in serum-free media (Opti-MEM) ± test drugs for the indicated times at 37 °C. Cells were then washed with warm binding buffer (10 mm HEPES, 135 mm NaCl, 5 mm KCl, 1 mm CaCl_2_, 1 mm MgCl_2_, pH 7.4) and incubated in binding buffer at 37 °C for 10 min to remove excess drug. Cells were washed with ice-cold binding buffer and incubated in 3 nm [^3^H]granisetron (in binding buffer) for 120 min on ice. Excess radioligand was removed by washing with ice-cold binding buffer. Receptor-bound radioligand was then eluted by the addition of acidic saline (0.2 m acetic acid, 0.5 m NaCl, pH 2.5) for 30 min, added to scintillation mixture, and counted. Background (mock) binding was subtracted, and specific binding was expressed as a percentage of untreated cells.

##### Cell Surface ELISA

Methods were as previously described (22). Transiently transfected COS-7 cells were grown on poly-l-lysine-coated 96-well plates. Cells were incubated in Opti-MEM ± 5-HT at the indicated concentrations for 60 min at 37 °C. All subsequent solutions were made in PBS (137 mm NaCl, 2.7 mm KCl, 10 mm Na_2_PO_4_, 1.8 mm KH_2_PO_4_, 1 mm CaCl_2_, 1 mm MgCl_2_, pH 7.4) unless stated otherwise. After experimental treatment, cells were fixed in ice-cold 3% paraformaldehyde (in PBS) for 15 min and then washed with PBS. To reduce background signal, cells were incubated in 0.1 m glycine for 60 min, washed, and then incubated in 3% H_2_O_2_ (to quench endogenous peroxidase activity) for 5 min, washed again, and then blocked for 60 min (5% FBS and 1% BSA in PBS). Primary antibody (anti-HA; 12CA5) was incubated for 60 min and then washed 3 times with block. Secondary antibody (sheep anti-mouse HRP) diluted to 400 μg/ml in block) was incubated for 60 min and subsequently washed 3 times in PBS. Cells were then incubated with Amplex UltraRed (3 μm) and H_2_O_2_ (740 μm) for 60 min in the dark, and the plate was read at excitation/emission wavelengths of 530/590 nm. Background (mock) values were subtracted, and surface expression levels were presented as percentage of untreated cells.

##### Receptor Internalization Assay

COS7 cells were prelabeled with mouse anti-HA antibodies (Sigma) (60 min, on ice), washed, and incubated in the absence or presence of 5-HT (300 μm) for 60 min at 37 °C. Cells were fixed in 3% paraformaldehyde (in PBS) and washed twice in 50 mm NH_4_Cl (in PBS) and blocked (10% FBS, 0.5% BSA in PBS) for 30 min. Subsequent washes and antibody dilutions were performed in PBS containing 10% FBS and 0.5% BSA. Surface receptors were detected using anti-mouse Alexa Fluor 488 (Molecular Probes). Cells were then permeabilized by the addition of 0.5% Triton X-100 (10 min), and the immunofluorescence protocol was repeated from the NH_4_Cl step using anti-mouse Alexa Fluor 568 to detect internalized receptors. Cells were examined using a Hamamatsu ORCA-ER camera mounted on a DM-IRB inverted microscope using Volocity software (PerkinElmer Life Sciences).

##### 5-[^3^H]HT Uptake Assay

COS-7 cells transfected with human SERT or mock-transfected cells were grown on 24-well plates. Cells were washed with uptake buffer (10 mm HEPES, 135 mm NaCl, 5 mm KCl, 10 mm glucose, 100 μm ascorbic acid, 1 mm CaCl_2_, 1 mm MgCl_2_, pH 7.4) then incubated with 5-[^3^H]HT (as indicted) for 60 min at 37 °C. Cells were washed twice with ice-cold buffer, solubilized with 1% TX-100, then added to scintillation mixture and counted.

##### TX-114 Phase-partitioning of 5-[^3^H]HT

Transfected COS-7 cells were grown in 24-well plates. Cells were incubated with Opti-MEM containing 10 μm 5-HT (1:10 ratio 5-[^3^H]HT:cold 5-HT) for 60 min at 37 °C. Cells were washed (3 times) to remove surface receptor-bound 5-HT. Cells were then solubilized with 1% TX-114 (5 min on ice). Nuclei were pelleted by centrifugation (14,000 rpm, 5 min, 4 °C), the post-nuclear supernatant was warmed (5 min, 37 °C) to precipitate the detergent fraction-containing membranes and centrifuged (14,000 rpm, 5 min, 20 °C), and the soluble fraction (cytosol) was removed and counted in scintillation fluid. The membrane fraction was washed (1% TX-114) on ice to re-solubilize the detergent/membrane fraction. This process was repeated twice to wash the membrane fraction before being counted. All intermediate washes (soluble fraction) were pooled and counted. Background counts (scintillation fluid only) were subtracted.

##### Measurement of Cytoplasmic 5-[^3^H]HT

Transfected COS-7 cells were grown in 24-well plates. Cells were incubated with Opti-MEM containing 10 μm 5-HT (1:10 ratio 5-[^3^H]HT:cold 5-HT) for 60 min at 37 °C. To remove surface receptor-bound 5-HT, cells were washed with warm binding buffer and then once with ice-cold buffer. Cells were incubated with 100 μg/ml digitonin (diluted in binding buffer) for 5 min, and the buffer was removed to count 5-[^3^H]HT associated with the cytoplasmic (released) contents. Cells were washed twice with ice-cold binding buffer (and each wash counted) and then solubilized with 1% TX-100 to count radioactivity remaining within the cells.

##### Contractile Responses in the Guinea Pig Ileum

Male or female Dunkin-Hartley guinea pigs (500–1000 g) were sacrificed by cervical dislocation. The ileum was removed, and sections of tissue 15–25 mm in length were suspended in organ baths containing 10 ml of air-bubbled Tyrode buffer (137 mm NaCl, 2.7 mm KCl, 1.5 mm CaCl_2_, 1 mm MgCl_2_, 12 mm NaHCO_3_, 0.4 mm NaH_2_PO_4_, 28 mm glucose, pH 7.4) maintained at 37 °C. Isotonic responses were measured using a force transducer, and a resting tension of 1 g was applied. Responses to a maximal concentration (50 μm) of the 5-HT_3_ receptor agonist, 2-methyl 5-hydroxytrypamine (2-ME) were measured to determine the basal 5HT_3_-mediated contraction of each tissue. Before receptor desensitization, tissue was washed and allowed to recover (10 min, to ensure receptor resensitization) before further stimulation. For chronic exposure, 100 μm 5-HT was applied for 60 min and washed out (10 min), and subsequent 2-ME responses were measured. To determine the specificity of the responses, contractions evoked by acetylcholine (ACh) (1 μm) were monitored. In acute 5-HT exposure experiments, guinea pig ileum tissue was exposed to 100 μm 5-HT until receptors desensitized (contractile response returned to base line) before recover experiments. Recovery after 10 min (±5 μm 5-HT) was tested using 2-ME (50 μm). All drugs (apart from the experimental chronic 5HT treatment) were washed out immediately after peak responses were observed. The peak response to each drug addition was expressed as a percentage of the peak response at the beginning of the experiment.

##### 5-HT Release Assay

Cos-7 cells were plated onto polylysine-coated 96-well plates and grown for 24–48 h. Cells were treated with 100 μl of 300 μm 5-HT (0.1% 5-[^3^H]HT, specific activity = 27.7 Ci/mmol) for 1 h at 37 °C in Opti-MEM and washed twice with 100 μl of warm Opti-MEM and left at 37 °C for the times indicated. Samples (100 μl) were placed into a scintillation mixture, and radioactivity was counted. Segments of adult guinea pig ileum were treated with 100 μm 5-HT (0.1% 5-[^3^H]HT) for 1 h in air-bubbled Tyrode buffer. After treatment segments were briefly washed and stored in Tyrode buffer (1.5 ml) for the indicated times, and samples (100 μl) were placed into a scintillation mixture for counting.

## RESULTS

### 

#### 

##### Chronic Exposure to 5-HT Decreases Cell Surface 5-HT_3_ Receptor Binding Sites in an Endocytosis-independent Manner

**FIGURE 1.**
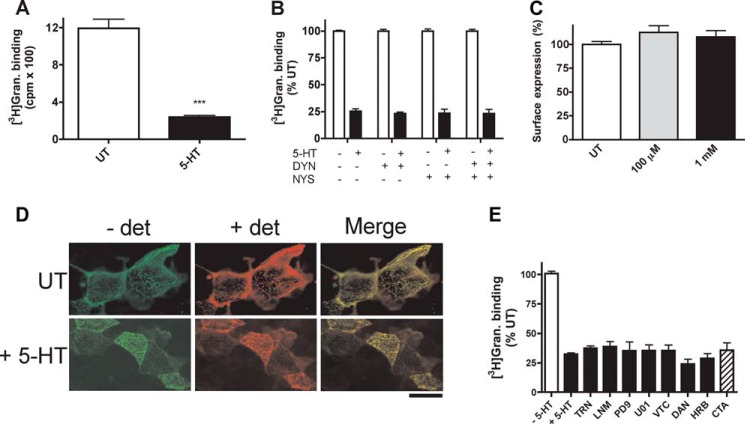
**Chronic exposure to 5-HT decreases 5-HT_3_ receptor [^3^H]granisetron binding sites in COS-7 cells through endocytosis-independent mechanisms.** *A*, binding sites measured after chronic 5-HT exposure (300 μm, 60 min, 37 °C) or untreated control (*UT*). Data represent an average of five experiments performed in triplicate. *** signifies *p* < 0.001, paired *t* test. *B*, normalized (to untreated) binding sites measured after chronic 5-HT exposure (300 μm, 60 min, 37 °C) in the presence of 80 μm dynasore (*DYN*), 21 μm nystatin (*NYS*), and both dynasore and nystatin. Data represent an average of three experiments performed in triplicate. *C*, ELISA measuring cell surface expression of 5-HT_3_A-HA after chronic exposure to 5-HT (300 μm or 1 mm, 60 min, 37 °C). Data represent an average of three experiments performed in triplicate. *D*, immunofluorescence detection of surface (no detergent (− *det*)) receptors in the absence (−5-HT) or presence (+ 5-HT) of 5-HT. *E*, the effect of key signaling pathways on down-regulation investigated in 5-HT3A and SERT co-expressing cells. Cells were incubated (60 min, 37 °C) with no drugs or 5-HT (−*5-HT*), no drug with 300 μm 5-HT (+*5-HT*), or one of the following drugs (present before (60 min, 37 °C) and during 5-HT (300 μm, 60 min) treatment): cysteamine (*CTA*, 500 μm), dansylcadaverine (*DAN*, 200 μm), vitamin C (*VTC*, 50 μm), tiron (*TRN*, 5 mm), PD98059 (*PD9*, 10 μm), UO126 (*UO1*, 10 μm), herbimycin A (*HRB*, 2 μm) or L-NAME (*N*^G^-nitro-l-arginine methyl ester (*LNM*), 1 mm). Cells were washed (10 min, 37 °C) before monitoring the remaining binding sites with [^3^H]granisetron. Data represent an average of three or four experiments performed in triplicate.

The effect of chronic exposure of 5-HT_3_ receptors to 5-HT was investigated in COS-7 cells expressing human 5-HT_3_A receptors. Cells were incubated with 5-HT (300 μm, 60 min, 37 °C, followed by 10 min washout), and the remaining 5-HT_3_ receptor binding sites were quantified by [^3^H]granisetron binding (3 nm, 120 min, 4 °C). Under these experimental conditions we measured only cell surface receptor binding sites (22). We found that the chronic exposure to 5-HT causes a significant decrease in receptor binding (*p* < 0.001; paired *t* test; *n* = 5) to 20.5 ± 4.0% of untreated cells (Fig. 1*A*).

A potential mechanism for 5-HT-mediated 5-HT_3_ loss of function is the promotion of receptor internalization (17, 18). To investigate this we used inhibitors of clathrin- and caveolin-mediated endocytosis. 5-HT_3_A-expressing cells were preincubated (60 min, 37 °C) with either dynasore (80 μm) a clathrin inhibitor, nystatin (21 μm), a caveolin inhibitor, or both together. Cells were then exposed to 5-HT (300 μm) in the presence of the inhibitors (60 min, 37 °C). Subsequent binding sites were then measured using [^3^H]granisetron and expressed as the percentage of binding in control cells (exposed to the inhibitors but not 5-HT). Neither dynasore, nystatin, nor both together block the loss of function caused by prolonged exposure to 5-HT (*p* > 0.05; ANOVA; Fig. 1*B*), suggesting that neither clathrin- nor caveolin-mediated endocytosis is required for the reduction in binding sites observed.

To confirm a lack of involvement of receptor internalization, we monitored cell surface receptor levels using an extracellular epitope (HA) tag and whole cell ELISA. 5-HT_3_A-HA receptor-expressing cells were incubated in the presence/absence of 5-HT (60 min, 37 °C) and fixed (3% paraformaldehyde) to prevent further membrane trafficking, and the remaining surface receptors were quantified by ELISA. Exposure to 5-HT (100 μm or 1 mm) had no effect on cell surface receptor levels (112.6 ± 12.4 and 107.7 ± 11.8%) compared with untreated cells (100 ± 15.5%), respectively (*p* > 0.05; ANOVA, *n* = 3; Fig. 1*C*). Clearly, the cell surface 5-HT_3_ receptor levels remained stable during chronic exposure to high levels of 5-HT despite the presence of a robust loss of surface binding sites (Fig. 1*A*). To further demonstrate that receptor internalization does not occur, we performed a fluorescence endocytosis assay to track surface receptors. Cell surface receptors were labeled with an anti-HA antibody (on ice) and then incubated (60 min, 37 °C) in the presence of absence of 5-HT (300 μm, 60 min). Receptors remaining on the cell surface were identified using an Alexa 488-tagged secondary antibody (− *det*), cells were then permeabilized and reprobed using an Alexa 568 tagged secondary antibody (+ *det*) to identify internalized receptors (Fig. 1*D*). In support of the ELISA and use of endocytosis inhibitors, no receptor internalization was observed.

A number of signaling events mediated by intracellular 5-HT have been identified previously. These include serotonylation (transglutaminase-mediated attachment of 5-HT to target proteins) (29, 30), the formation of reactive oxygen species accompanied by ERK1/2 MAPK and tyrosine kinase activation (31), and the regulation of nitric oxide signaling (32). To investigate potential roles for these events in the regulation of 5-HT_3_ receptors, a range of inhibitors was used. 5-HT_3A_-expressing cells were preincubated for 60 min at 37 °C with each inhibitor, and 5-HT (300 μm) induced down-regulation monitored and related to inhibitor only (no 5-HT) controls. The transglutaminase inhibitors cysteamine (*CTA*, 500 μm) or dansylcadaverine (*DAN*, 200 μm), the antioxidants (used to inhibit reactive oxygen species) vitamin C (*VTC*, 50 μm) or tiron (*TRN*, 5 mm), the ERK1/2 inhibitors PD98059 (10 μm) or UO126 (10 μm), the tyrosine kinase inhibitor herbimycin A (2 μm), or the nitric oxide synthase (*NOS*) inhibitor L-NAME (*N*^G^-nitro-l-arginine methyl ester, *LNM*, 1 mm) were without effect on the ability of 5-HT to down-regulate 5-HT_3_ binding sites (Fig. 1*E*; *p* > 0.05, ANOVA, *n* = 4). These findings indicate that intracellular 5-HT signaling via previously identified pathways is not required for 5-HT_3_ receptor loss of function.

##### Down-regulation of 5-HT_3_ Receptor Function in the Guinea Pig Ileum

**FIGURE 2.**
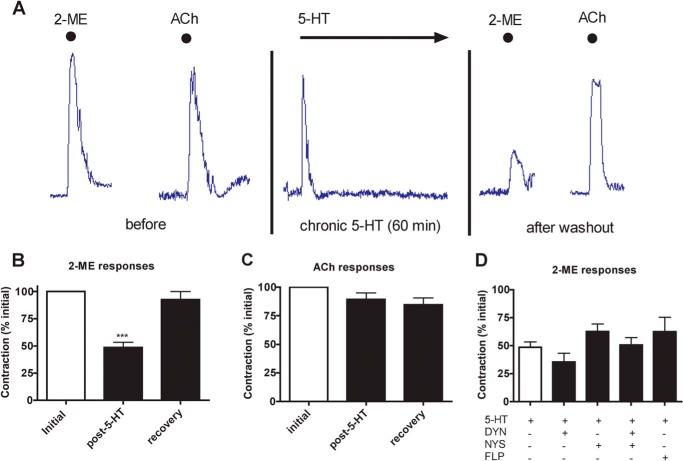
**5-HT_3_ down-regulation of function in the guinea pig ileum occurs through endocytosis-independent mechanisms.** *A*, sample trace of an organ bath experiment investigating the chronic effects of 5-HT. *Dots* represent brief exposure times whereby the indicated drug was washed out immediately after maximal contraction obtained. The *arrow* indicates prolonged (60 min) exposure with no washout. *B*, the contractile responses to 2-ME (50 μm) were measured in the tissue (*Initial*). After 60 min of exposure to 100 μm 5-HT (followed by 10 min washout), the resultant 2-ME responses were measured (*post-5-HT*). 2-ME responses were also tested 60 min post treatment to monitor long term recovery. Data represent an average of 28 independent tissue samples. *** signifies *p* < 0.001 (ANOVA). *C*, responses to 1 μm ACh before and after 5-HT treatment (60 min) and after a 60-min recovery in drug-free buffer. Data represent an average of 14 sections of tissue. *D*, tissue responses to 2-ME (50 μm) after chronic exposure to 5-HT (100 μm, 60 min) in the absence and presence of dynasore (*DYN*; 80 μm), nystatin (*NYS*; 21 μm), dynasore plus nystatin, or filipin (*FLP*; 7.6 μm). Data are an average of 8–33 sections of ileum.

As high levels of 5-HT are observed within the gut, we investigated whether chronic exposure to 5-HT led to a ligand-induced down-regulation of receptor function in the intact guinea pig ileum. Specific 5-HT_3_ receptor activation was monitored by the contractile responses induced by the selective agonist 2-ME. Acute exposure to 5-HT (100 μm) caused a robust contraction and rapid receptor desensitization (Fig. 2*A*, *center trace*) which recovered quickly after washing (<10 min, not shown). In contrast, chronic exposure to 5-HT (100 μm, 60 min), followed by washout in a drug-free buffer for 10 min (to allow receptor resensitization) caused a significant decrease (to 48.6 ± 25.0%, Fig. 2*B*) in the magnitude of subsequent 2-ME-evoked contractions (*p* < 0.001, ANOVA, *n* = 28) (Fig. 2*A*, *right trace*) compared with the pretreatment contraction (Fig. 2*A*, *left trace*). After removal of 5-HT (60 min) the responses to 2-ME were fully recoverable (Fig. 2*B*). To investigate the specificity of the 5-HT_3_ functional loss, contractions evoked by ACh were also quantified. In this case the magnitude of ACh responses were unaffected by chronic 5-HT treatment (*p* > 0.05; ANOVA, *n* = 14; Fig. 2*C*), verifying that the tissue was still capable of undergoing contractions and that the down-regulation was specific to 5-HT_3_ receptors. An individual example of 5-HT_3_ functional loss is shown (Fig. 2*A*) where a pre-pulse of 2-ME and ACh precedes 5-HT chronic (60 min) exposure (note early receptor desensitization). After the chronic exposure to 5-HT, 2-ME, but not ACh, responses were reduced.

To determine whether receptor internalization contributed to the loss of 2-ME responsiveness in the ileum, dynasore (80 μm) or nystatin (21 μm) were co-applied with 5-HT during the chronic exposure (60 min). Neither drug alone or in combination had a significant effect on 5-HT_3_ contractions or the subsequent loss of responsiveness (*p* > 0.05; ANOVA, *n* = 8- 33; Fig. 2*D*). Similarly, filipin (7.6 μm), an additional caveolin inhibitor, also failed to prevent the loss of 5-HT-mediated loss of responsiveness (Fig. 2*D*). Together, these results suggest that receptor internalization via clathrin- or caveolae-dependent pathways is not responsible for the dynamic changes in 5-HT_3_ receptor-induced contractions that we observe in intact ileum (Fig. 2) or loss of binding sites in COS-7 cells (Fig. 1).

##### 5-HT_3_ Down-regulation of Function Is Potentiated by SERT

**FIGURE 3.**
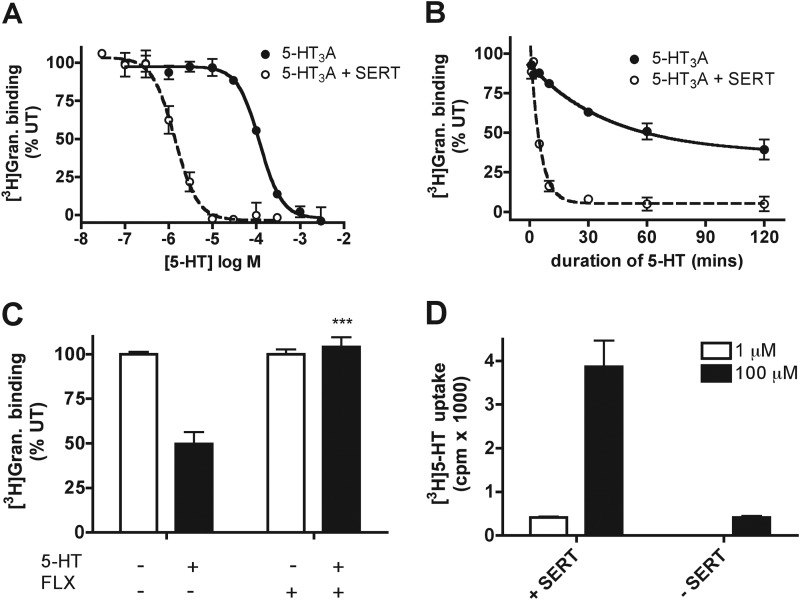
**SERT potentiates 5-HT-mediated down-regulation of 5-HT_3_ receptor function in COS-7 cells.** *A*, concentration-response to [^3^H]granisetron (*Gran*) binding site availability after chronic 5-HT exposure (60 min, 37 °C) to cells expressing 5-HT_3_A (*filled circles*, *solid line*) or 5-HT_3_A and SERT (*open circles*, *dashed line*). Data represent an average of 3–4 experiments and are fitted to a sigmoidal dose-response curve (variable slope) function. *UT*, untreated control. *B*, time-course of 5-HT (100 μm) induced down-regulation in 5-HT_3_A (*filled circles*, *solid line*) or 5-HT_3_A and SERT-expressing cells (*open circles*, *dashed line*). Data represent the average of three experiments performed in triplicate and are plotted using the one-phase exponential decay function. *C*, [^3^H]granisetron binding sites measured after chronic exposure to 5-HT (3 μm, 60 min, 37 °C) in the absence (*UT*) or presence of fluoxetine (*FLX*, 10 μm) in 5-HT_3_A and SERT co-expressing cells. *** signifies *p* < 0.05; Mann Whitney test. *D*, 5-HT (5-[^3^H]HT) uptake into COS-7 cells in the absence (−*SERT*) or presence (+*SERT*) of SERT expression.

As extracellular 5-HT is rapidly sequestered by the SERT transporter, we investigated whether this loss could be influenced by the cellular influx of 5-HT. Therefore, we investigated the impact of human SERT co-expression with the receptor (equimolar cDNA ratios). Cells were incubated with a range of concentrations of 5-HT (60 min, 37 °C), and the remaining surface receptor binding sites were determined by [^3^H]granisetron binding (Fig. 3*A*). Regardless of the presence of SERT, chronic 5-HT exposure caused a concentration-dependent decrease in receptor binding sites. Cells expressing 5-HT_3_A alone required a high concentration to cause a subsequent inhibition of binding, with an IC_50_ of 154.0 ± 45.7 μm, and complete loss was observed at ∼1 mm 5-HT. In contrast, cells co-expressing SERT are more sensitive to 5-HT exposure, exhibiting an IC_50_ of 2.3 ± 1.0 μm (a 67-fold enhancement by SERT), and complete inhibition occurred at ∼10 μm (Fig. 3*A*).

As SERT increases the potency of 5-HT-induced down-regulation, we also investigated its effect on the kinetics of functional loss. Cells expressing 5-HT_3_A in the presence or absence of SERT were exposed to 100 μm 5-HT, and surface binding sites were monitored over time with [^3^H]granisetron (Fig. 3*B*). A time-dependent reduction in receptor binding sites were observed, with a markedly faster rate was observed in SERT-expressing cells (*t*_½_ = 3.4 min) compared with those without SERT (*t*_½_ = 28.6 min). Taken together, these findings indicate that the loss of surface receptor binding sites is related to the level of cellular uptake of 5-HT.

##### SERT Function Is Required for Its Potentiating Effect on 5-HT-mediated Loss of Function

Given the potentiation of down-regulation by SERT, we investigated whether SERT function is required. 5-HT_3_A and SERT co-expressing cells were preincubated with the SERT inhibitor, fluoxetine (10 μm, 10 min, 37 °C), before exposure to 5-HT (3 μm, 60 min, 37 °C) in the continued presence of fluoxetine (Fig. 3*C*). In keeping with a requirement 5-HT influx, fluoxetine completely blocked the loss of function (103.98 ± 10.84% of untreated, *p* < 0.05; Mann Whitney test). In contrast, in cells in which SERT was not expressed, fluoxetine did not block down-regulation to 5-HT (1 mm) (not shown).

To confirm 5-HT cellular uptake, we monitored 5-[^3^H]HT accumulation. Cells were incubated with 5-[^3^H]HT (1 or 100 μm) for 60 min at 37 °C and washed, and cell-retained 5-[^3^H]HT was counted. As expected, SERT-expressing cells demonstrated a robust concentration-dependent uptake of 5-[^3^H]HT (Fig. 3*D*). In contrast, in cells lacking SERT expression, 5-[^3^H]HT uptake was only detected at 100 μm (Fig. 3*D*). The concentration-dependent effects (IC_50_ = 2.3 (+ SERT) or 154 μm (−SERT) (Fig. 3*A*) on down-regulation are consistent with a requirement for intracellular 5-HT transport.

##### Cytoplasmic 5-HT Is Required to Inhibit 5-HT_3_ Receptor Binding

**FIGURE 4.**
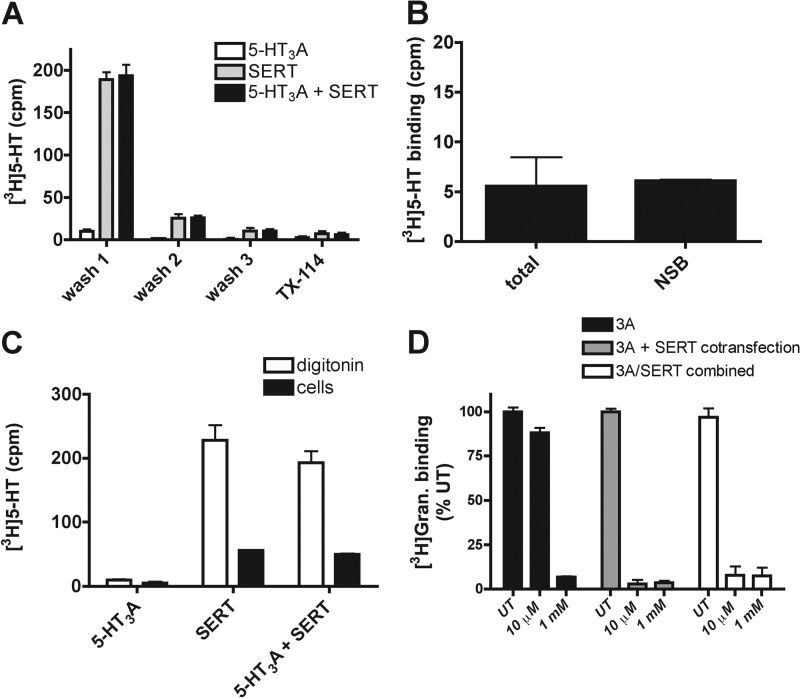
**Cytosolic accumulation of 5-HT correlates with the loss of receptor binding sites.** *A*, cells expressing 5-HT_3_A, SERT, or 5-HT_3_A + SERT were loaded with 10 μm 5-HT (10% 5-[^3^H]HT) for 60 min at 37 °C. Postnuclear cytosolic (TX-114 supernatant at 37 °C) fractions were counted (*wash 1*). The membrane pellet washed (*wash 2* and *3*) and remaining membrane pellet fraction (*TX-114*) were counted. Data represent an average of three experiments performed in triplicate. *B*, 5-HT_3_A-expressing cells were incubated with 10 μm 5-[^3^H]HT with (nonspecific binding (*NSB*)) or without (*total*) 10 μm ondansetron (60 min at 37 °C). *C*, cells expressing 5-HT_3_A, SERT, or both were incubated with 10 μm 5-HT (10% 5-[^3^H]HT) for 60 min at 37 °C. Cells were permeabilized with digitonin (100 μg/ml) to release cytosolic 5-HT and compared with cell-associated signal. Data represent an average of three experiments performed in triplicate. *D*, cells expressing 5-HT_3_A only (*3A*), 5-HT_3_A and SERT (*3A* + *SERT cotransfection*), or cells expressing either 5-HT_3_A or SERT and subsequently mixed (*3A/SERT combined*) were examined for 5-HT (10 μm or 1 mm, 60 min, 37 °C)-induced down-regulation of receptor binding sites ([^3^H]granisetron (*Gran*)). *UT*, untreated.

To gain further insight into a potential mechanism of action, we investigated whether transported 5-[^3^H]HT was associated with cellular membranes or remained soluble within the cytoplasm. Cells were incubated with 5-HT (10 μm, 10% of which was 5-[^3^H]HT) in Opti-MEM for 60 min at 37 °C, and cells were washed with binding buffer before being solubilized with 1% TX-114 (5 min, 4 °C). Radioactivity associated with the membrane phase and each sequential wash (cytosol) was counted (Fig. 4*A*). In keeping with previous observations, where cells lacking SERT exhibited little cellular uptake (Fig. 3*D*) and no loss of function (Fig. 3*A*) at this concentration of 5-HT, we found no significant intracellular 5-[^3^H]HT accumulation (Fig. 4*A*). In SERT-expressing cells (regardless of 5-HT_3_ receptor expression), robust counts were detected in the first cytosolic fraction (*wash 1*), with diminishing counts associated with subsequent washes. Minimal 5-[^3^H]HT remained in the final TX-114 membrane fraction, suggesting that intracellular 5-HT is predominantly freely soluble. Importantly, in cells co-expressing SERT and 5-HT_3_A receptors, no increased cellular retention or membrane association was observed. Therefore, prolonged receptor association through a putative intracellular binding site is not responsible. Moreover, in our assays, 5-HT (10 μm) does not exhibit prolonged 5-HT_3_ receptor binding, as no 5-[^3^H]HT binding (Fig. 4*B*, *total*) was observed above the nonspecific binding (as determined by competition with excess ondansetron) after rapid washing in 5-HT_3_A-transfected cells.

To confirm that SERT-mediated 5-HT accumulation is readily releasable from cells, we used digitonin to permeabilize the cells and release the cytoplasmic contents. Cells were loaded with 10 μm 5-HT as above, and excess 5-HT was removed. Cells were then chilled on ice and incubated with digitonin (100 μg/ml in binding buffer) for 5 min on ice. The released (3 consecutive washes pooled) and cell-associated (solubilized in 1% TX-100) 5-[^3^H]HT was counted. In cells expressing 5-HT_3_A receptors alone, no detectable 5-HT accumulation was detected (Fig. 4*C*) consistent with previous experiments. In cells expressing SERT alone, the majority of counts (79.1 ± 3.3% total) were detected in the cytoplasmic phase. Similarly, in cells co-expressing SERT and 5-HT_3_A receptors, 80.0 ± 3.0% of total counts were in the cytoplasmic phase. Although a proportion of the 5-HT remains associated with the cells, this is not bound to the receptors, as the levels of cell-associated counts are not increased by 5-HT_3_ receptor expression.

To distinguish between a cytoplasmic role for 5-HT from any consequential effects following its subsequent release from cells (extracellular effects), we investigated whether the co-expression of SERT within the same cell is required. Therefore, we compared the down-regulation responses to low 5-HT (10 μm) in a population of cells co-expressing the receptor and transporter with a mixture of two populations of cells, one expressing the receptor only and the other expressing only the transporter. A cytoplasmic function of 5-HT would be potentiated by SERT only if it is co-expressed on the same cells. In contrast, an extracellular effect would also occur if SERT expression was limited to neighboring cells.

As shown previously (Fig. 3*A*), when 5-HT_3_ and SERT are co-expressed, low 5-HT (10 μm) is sufficient to induce down-regulation (Fig. 4*D*, *3A* + *SERT cotransfection*). When independent cell populations expressing either 5-HT_3_ receptors or SERT were mixed after transfection (Fig. 4*D*, *3A/SERT combined*), the loss of binding sites was still induced by low 5-HT. This indicates that the potentiation of down-regulation seen by SERT does not require 5-HT transport into the same cells expressing the 5-HT_3_ receptors. Therefore, the cytoplasmic pool of 5-HT may provide a reservoir from which subsequent 5-HT release may influence 5-HT_3_ receptor function.

##### 5-HT Release and Inhibition of Receptor Resensitization

**FIGURE 5.**
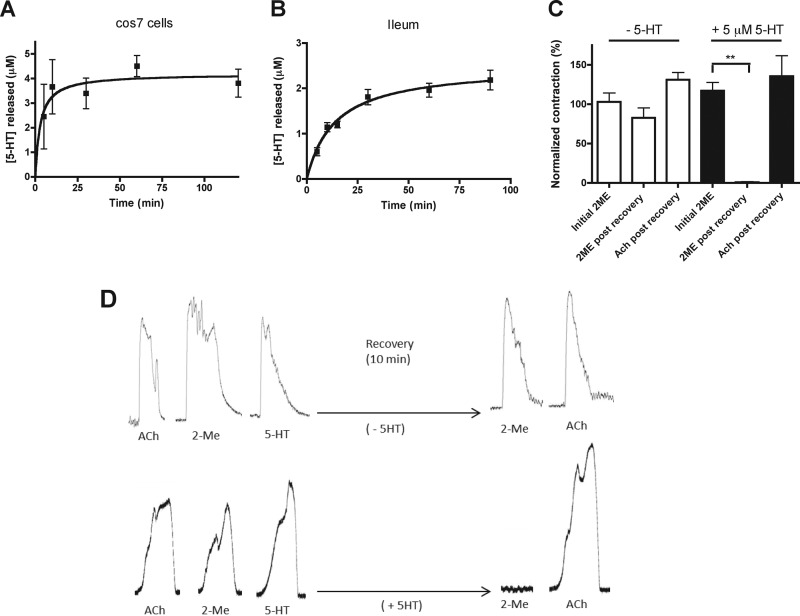
**5-HT release and its impact on receptor resensitization.** *A*, time course of 5-HT release from COS-7 cells after loading (300 μm 5-HT (0.1% 5-[^3^H]HT), 1 h). *B*, time course of 5-HT released from segments of adult guinea pig ileum following loading (100 μm 5-HT (0.1% 5-[^3^H]HT), 1 h). Data represent 20 segments of ileum from 4 different animals. *C*, 5-HT3 receptor resensitization in adult guinea pig ileum. Acute exposure to 5-HT (100 μm) was applied until full receptor desensitization observed and 5-HT_3_ receptor contractions (50 μm 2-ME) were determined after a 10-min resensitization period in the absence (−*5-HT*) or presence (+*5-HT*) of 5 μm 5-HT. Data represent 12 segments of ileum for each experiment on 3 separate occasions. *** signifies *p* = 0.0014 (paired *t* test, 2 tailed, *n* = 4). *D*, representative traces of guinea pig contractions indicating initial responses to ACh, 2-ME, and 5-HT (*left traces*) and subsequent 2-ME and ACh (*right traces*) responses after receptor desensitization to 5-HT (100 μm, <1 min) and subsequent recovery in the absence (*upper panel*) or presence (*lower panel*) of 5-HT (5 μm). Each *panel* (*upper* or *lower*) represents traces from a single continuous experiment.

To determine if cells are capable of releasing the sequestered 5-HT, we investigated the release of 5-[^3^H]HT from COS-7 cells and the guinea pig ileum. COS-7 cells (no SERT) were loaded with 5-HT (300 μm, 0.1% 5-[^3^H]HT, 60 min), excess 5-HT was washed off, and cells were chased for 120 min. Under the experimental conditions in which we observed down-regulation of function (Figs. 1 and 3), the release of 5-HT reached low micromolar levels (Fig. 5*A*). To determine if the same pool of 5-HT exists within cells in native tissue where high 5-HT level can occur, we investigated 5-HT uptake (300 μm, 0.1% ^3^H-5-HT, 60 min) in guinea pig ileum segments. A 7-cm segment was turned inside out and incubated with 5-HT (100 μm, 0.16% ^3^H-5-HT, 60 min). Five equal sections were cut, and 5-HT release was monitored (in 1.5 ml) from each. Under these conditions, 5-HT is sequestered and released from the ileum in the low micromolar range (Fig. 5*B*).

In COS-7 cells expressing SERT, 5 μm 5-HT was sufficient to cause significant loss of function (IC_50_ = 2.3). However, in the absence of SERT it exerted little effect (IC_50_ 154 μm) on 5-HT_3_ receptor function (Fig. 3*A*). Therefore, we investigated whether sustained low concentrations of 5-HT might inhibit receptor resensitization. Therefore, guinea pig ileum segments were first validated for contractile responses (1 μm ACh) (not shown), and the magnitude of 2-ME (50 μm) responses was determined. After recovery to base line, 5-HT (100 μm) was then applied until full desensitization occurred. Recovery from desensitization of 5-HT3 receptors was then monitored (50 μm 2-ME) after 10 min in the absence of 5-HT, where recovery (82.5 ± 21.8%) occurred (Fig. 5, *C* and *D*). However, in the presence of 5-HT (5 μm), no recovery (0.9 ± 0.9%) occurred (*p* = 0.0014, paired *t* test, 2 tailed, *n* = 4). Therefore, chronic exposure to high levels of 5-HT leads to receptor desensitization and local 5-HT sequestration followed by a subsequent slow release of 5-HT that inhibits 5-HT3 receptor resensitization.

## DISCUSSION

Dynamic ligand-induced regulation of 5-HT_3_ receptors is likely to have relevance to a number of (patho)physiological situations characterized by changes in 5-HT levels. Here we report that with recombinantly expressed human 5-HT_3_ receptors and in the intact guinea pig ileum, chronic exposure to 5-HT caused a transient down-regulation of receptor function. In both cases we showed that this loss occurs independently of receptor endocytosis. Intriguingly, we found that intracellular 5-HT sequestration plays an important role, and this was supported by potentiation by SERT. However, this was not due to serotonylation or known signaling pathways. These findings offer a novel regulatory mechanism by which 5-HT_3_ receptors may be modulated by their endogenous ligand after circumstances of chronic exposure.

Agonist-mediated down-regulation of 5-HT_3_ receptors has been reported in a number of settings (17, 21). We corroborate these findings by demonstrating that chronic exposure to 5-HT can reduce 5-HT_3_ receptor binding sites in a recombinant expression system and mediate a loss of 5-HT_3_ receptor-induced contractility in the intact ileum.

Previous reports have suggested a reduction in cell surface receptor numbers through internalization as a mechanism for agonist-mediated down-regulation (17, 18). However, we directly tested the role of receptor endocytosis and present clear evidence that down-regulation is insensitive to inhibitors of endocytosis and demonstrated that no internalization or changes in cell surface receptor levels occur.

It is possible that species difference is responsible for the distinct findings observed previously. We have investigated human (recombinant) and guinea pig receptors, whereas previous studies have focused on mouse (18) or rat (17) receptors. Similarly, we have used COS-7 cells or intact ileum, whereas HEK293 cells (18) or enteric neurons (17) were investigated previously. However, much of the data presented by both groups can be explained by a loss of receptor binding sites. In one study (18), 5-HT was not used as the agonist, and ligand-specific effects are possible.

An alternative mechanism is slowed dissociation kinetics of 5-HT. Indeed we have demonstrated recently that the clinically used 5-HT_3_ receptor antagonists palonosetron and ondansetron chronically down-regulate 5-HT_3_ receptors via prolonged receptor binding (22). However, we do not find prolonged interactions of 5-HT with receptors or any other cellular targets. Indeed, relative to its acute effects (EC_50_ ∼ 6 μm) (48) at 5-HT_3_A receptors, supramaximal concentrations of 5-HT (IC_50_ 154 μm) are required to produce down-regulation (in the absence of SERT expression), suggesting a mechanism of action independent of its acute binding to the orthosteric receptor binding site. However, when cellular uptake of 5-HT is increased by SERT coexpression, the potency and kinetics of 5-HT-induced down-regulation is increased accordingly. Therefore, contrary to previous interpretations and alternative mechanisms, we propose that during periods of high 5-HT levels, cellular sequestration occurs, and this provides a reserve pool of 5-HT from which a constant unregulated release occurs. By mimicking our experimental release of 5HT observed during our cellular studies, we show that low levels of 5-HT prevent recovery from receptor desensitization of 5-HT3 receptors in the guinea pig ileum.

Therefore, we propose that low levels of 5-HT may cause receptor desensitization (33) but not down-regulation, as insufficient 5-HT is sequestered, and receptors recovery rapidly on the removal of 5-HT. In contrast, at higher levels or in the vicinity of SERT expression, 5-HT sequestration may be sufficient to result in a prolonged release of 5-HT at low level that is sufficient to prevent receptor resensitization. 5-HT release plateaus in the closed large volume systems used here (COS-7 cells or guinea pig ileum) suggesting an equilibrium is reached. The physiological relevance of our observations of 5-HT-mediated regulation of 5-HT_3_ receptors will depend on the duration and concentration of endogenous 5-HT exposure, the local expression of SERT, and the opportunity for diffusion away from the site of release. Under basal conditions, 5-HT release from the rat ileum is ∼6 μm, peaking to 16 μm upon mechanical stimulation (34). These levels are increased somewhat when rats are fed a Western style high fat and high calorie diet (27). Plasma 5-HT concentrations in healthy individuals have been observed at tens of nanomolar, with increases of up to an order of magnitude in patients with irritable bowel syndrome with diarrhea, complex regional pain syndrome, depressive disorders, autism, or coronary artery disease (25, 26, 35, 36). Emesis caused by cancer therapy has been linked to increased 5-HT release from enterochromaffin cells in the gut (37). Moreover a robust and prolonged (over many hours) increase in 5-HT release from enterochromaffin cells linked to emesis has been reported after exposure to viral toxins (38). 5-HT_3_ receptor desensitization following a chronic 5-HT exposure event may protect the intestinal tract from mucositis as shown for receptor antagonism during 5-fluoruracil chemotherapy (39).

In the gut 5-HT_3_ receptors are postsynaptically expressed on sensory nerve endings and on interneurons of the enteric nervous system (40) and on non-neuronal enterochromaffin cells (41) where they make an important contribution to contraction. SERT is expressed in the mucosal epithelium as well as on serotonergic neurons but not on enterochromaffin cells (42, 43), and there is little direct evidence to suggest co-expression of 5-HT_3_ receptors and SERT within the same cells in the gut. However, we demonstrate that SERT co-expression in the same cells is not required and SERT-independent 5-HT uptake mechanisms exist (44, 45). Beyond the gut, 5-HT_3_ receptors and SERT are co-expressed within the same presynaptic cells in the solitary tract (46). Moreover, platelets, lymphocytes (B-cells), dendritic cells, glial cells, and sperm have also been reported to express both 5-HT_3_ receptors and SERT (46–49). Therefore, there is potential for 5-HT-mediated regulation of 5-HT_3_ receptors at physiologically relevant levels in the CNS, the blood, the immune system, and the reproductive system.

The pharmacotherapeutic use of selective serotonin reuptake inhibitors or the recreational use of drugs targeting SERT (such as cocaine, MDMA (3,4-methylenedioxy-*N*-methylamphetamine) and amphetamines) may also impact 5-HT_3_ desensitization by increasing 5-HT levels. To add further complexity to 5-HT_3_ receptor signaling, 5-HT can also regulate the function and expression of SERT or influence presynaptic autoreceptors (5-HT_3_/5-HT_4_) that modulate 5-HT release. Therefore, reciprocal cross-regulation of 5-HT_3_ and SERT may act to finely tune serotonergic signaling.

## Footnotes

2 The abbreviations used are:

5-HT_3_: 5-hydroxytryptamine type-3
SERT: serotonin transporter
TX: Triton X
2-ME: 2-methyl 5-hydroxytrypamine
ACh: acetylcholine
ANOVA: analysis of variance.
